# The ankle kinematic reference of normal gait pattern in Thai adults

**DOI:** 10.3389/fsurg.2022.915090

**Published:** 2022-08-11

**Authors:** Krongkaew Klaewkasikum, Tanyaporn Patathong, Chanika Angsanuntsukh, Thira Woratanarat, Jongsook Sanguantrakul, Patarawan Woratanarat

**Affiliations:** ^1^ Department of Orthopaedics, Faculty of Medicine Ramathibodi Hospital, Mahidol University, Bangkok, Thailand; ^2^ Department of Preventive and Social Medicine, Faculty of Medicine, Chulalongkorn University, Bangkok Thailand; ^3^ National Electronics and Computer Technology Center, National Science and Technology Development Agency, Pathumthani, Thailand

**Keywords:** motion analysis, foot progression, ankle rotation, gender, culture

## Abstract

**Objective:**

This study was aimed to establish the reference values of ankle kinematics and factors associated with ankle kinematics of healthy Thai adults.

**Methods:**

A prospective cohort was conducted among healthy volunteers aged between 18 and 40 years and evaluated gait analysis between 2016 and 2020. After applying the modified Halen Hayes marker set, participants were assigned to walk 8–10 rounds with their preferred speed. Demographic data i.e., age, gender and body mass index (BMI) and ankle kinematics (varus-valgus, dorsiflexion-plantar flexion, foot progression, and ankle rotation) using motion analysis software were recorded and analyzed.

**Results:**

98 volunteers (60 females and 38 males) aged 28.6 ± 5.4 years with body mass index 21.2 ± 2.0 kg/m^2^ were included. The average ranges of ankle kinematics entire gait cycle were varus-valgus −1.62 to 3.17 degrees, dorsiflexion-plantar flexion 0.67 to 14.52 degrees, foot progression −21.73 to −8.47 degrees, and ankle rotation 5.22 to 9.74 degrees. The ankle kinematic data in this study population was significantly different from the normal values supplied by OrthoTrak software of the motion analysis program, especially more ankle internal rotation at mid-stance (5.22 vs. −12.10 degrees) and terminal stance (5.48 vs. −10.74 degrees) with *P* < 0.001. Foot progression significantly exhibited more external rotation for 1.5 degrees on the right compared to the left side, and for 5 degrees more in males than females. One increment in age was significantly correlated with ankle internal rotation at mid-swing (coefficient 0.21 degrees, *P* = 0.039). BMI had no statistical association with ankle kinematics. Statistical parametric mapping for full-time series of angle assessments showed significantly different foot progression at initial contact and terminal stance between sides, and our ankle kinematics significantly differed from the reference values of the motion analysis program in all planes (*P* < 0.05).

**Conclusion:**

The reference of ankle kinematics of Thai adults was established and differences between sides and the normal values of the motion analysis program were identified. Advanced age was associated with ankle internal rotation, and male gender was related to external foot progression. Further studies are needed to define all-age group reference values.

## Introduction

The ankle joint mainly interacts with the ground to facilitate walking and performing activities (1). Its motions are varus-valgus occurring in the frontal plane; dorsiflexion-plantar flexion in the sagittal plane; foot progression and ankle rotation in the transverse plane (2, 3). The three-dimensional motion analysis system is widely used to evaluate ankle movements (4–8). This method requires a normal reference from healthy population to differentiate from abnormal conditions. Nevertheless, the reference value may not be universally applicable for every settings and populations (9).

Various references of ankle kinematics and possible contributing factors have been researched. Previous studies have proposed age, gender, culture (10), body shape, gait parameters (11), motion systems, settings (12), ethnic and geography (9, 11). Age and ethnics were primary factors related to ankle kinematics (9–11, 13–16). A systematic review and meta-analysis highlighted that advanced age diminished ankle motions, particularly in the sagittal plane (13). Elderly tended to have weaker plantar flexor leading to limited ankle moment at push-off than the younger age (14, 15). The best available evidence in Thai adults, aged 20–69 years, found ankle dorsiflexion-plantar flexion at toe off significantly reduced after age of 40 years (16). However, the authors investigated only the sagittal plane of ankle kinematics and did not compare with the standard reference of the motion system. Furthermore, they used the VICON system which cannot be applied to alternate motion analysis platforms.

Since the ankle kinematics varied among race, anthropometry, gait parameters, setting, and only sagittal plane was available among Thai adults, this study was aimed to investigate 3-dimension ankle kinematics in healthy Thai adults to provide better understanding our country-specific ankle kinematic pattern, and establish normative data as a reference of ankle joint complex in all dimensions. Even though the hips, knees, and ankles contributed to gait analysis, we would like to focus on the ankle joint to specify the reference values in all planes and compared with those of the motion analysis program. We included Thais, mostly indifferent geography, and our setting used the Motion Analysis program. This brought us to focus on age, gender, and BMI that could be related with the ankle kinematics and compared the reference values of the motion analysis program to ours. The results from this study would add the reference values not only ankle dorsiflexion-plantar flexion, but also ankle varus-valgus, foot progression, and ankle rotation; factors related with the ankle kinematics; and demonstrated how they differed from those of the motion analysis program. Moreover, the new reference values would help determining abnormal motion such as ankle instability, neuromuscular disorders, and deformity.

## Methods

A prospective cohort was conducted at Gait Laboratory, Department of Orthopaedics between 2016 and 2020. This study was approved by the Ethics Committee of Faculty of Medicine Ramathibodi Hospital (ID 06-59-26ว).

### Participants

Healthy Thai volunteers were included if their ages were between 18 years and 40 years in which presented the mature gait pattern; had a BMI between 18.5 and 25 kg/m²; and willing to participate in the study. This age range could avoid the toe-off reduction effect after 40 years old (16) and is commonly used to establish normal gait data (11, 17, 18). The exclusion criteria were having orthopedic problems within last 6 months; neuromuscular disorders; or loss of balance control.

### Procedures

**Figure 1:**
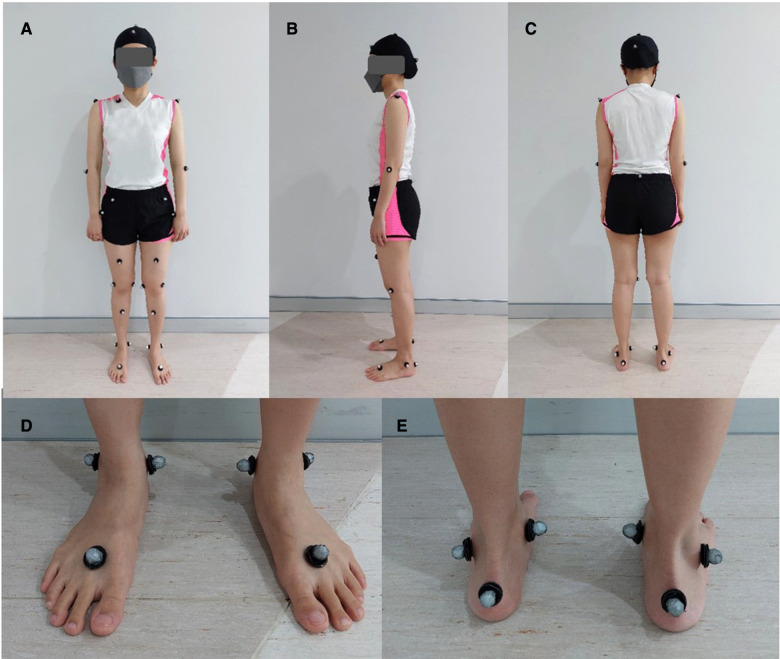
The modified Helen Hayes technique with 29 reflective markers included medial knees, (**A**) front, (**B**) side, and (**C**) back; and medial malleoli of the ankles, (**D**) front, and (**E**) back.

After providing informed consent, all volunteers underwent motion analysis. Regarding the modified Helen Hayes technique, 29 reflective markers were attached over participants' bony landmarks and both ankles, Figure 1 (18). All participants were asked to walk across 8 meters of the walkway, for 8–10 rounds with their preferred walking speed. Eight digital cameras captured all walking motion and Cortex software, Motion Analysis Corp, Santa Rosa, CA was used to track and process data. After ensuring the participants were familiar with their walking and all reflective markers were detected, the best 6 right, and 6 left gait cycles (2 cycles of 3 gait trials) were averaged and computed joint angle pattern for each participant. Gait velocity (m/s), stride length (m), and step length (m) were evaluated. Ankle kinematics in the frontal plane: ankle varus-valgus; the sagittal plane: dorsiflexion-plantar flexion; the transverse plane: foot progression, and also ankle rotation was assessed in degrees by motion analysis software (Orthotrak, Motion Analysis Corp).

### Data collection

We collected independent variables as gender, age, weight (kg), height (cm), body mass index; BMI (kg/m^2^), gait velocity, stride length, and step length, and dependent variables as the mean and standard deviation of ankle kinematics in all planes at heel strike, mid-stance, terminal stance, and mid-swing phases of the gait cycle. The ankle kinematic data was reported %gait cycle (0–100%) for each plane and defined as heel strike 0%–2%, mid-stance 12%–31%, terminal stance 31%–50%, and mid-swing 74%–87% of the gait cycle (19). The ankle kinematics were retrieved from the reference of the motion analysis program (20) for gait laboratories (OrthoTrak software, Motion Analysis Corporation, Santa Rosa, CA, USA) in order to compare with ours at a point of gait cycle i.e., heel strike, mid-stance, terminal stance and mid-swing.

### Statistical analysis

Baseline characteristics were presented as mean (standard deviation) for continuous variables and frequency (percentage) for categorical data. Ankle kinematics were summarized and compared between sides using paired t-test or Wilcoxon signed-rank test, in case of a non-normal distribution. Our ankle kinematic data was compared to the motion analysis reference using one-sample t-test or Wilcoxon signed-rank test. The relationship between age, gender, BMI and ankle kinematics was assessed by using linear regression reported as beta coefficient with 95% confidence interval (CI). Two-tailed tests with P-values  0.05 were considered statistically significant. Estimated sample size was 98, based on an alpha error of 0.05, beta error of 0.2, mean ankle dorsiflexion at mid-stance from a pilot study of 9.35 ± 1.2 degrees), and that mean from the previous study of Thai adults of 10.21 ± 3.16 degrees (16). All analysis was performed using STATA version 15.0 (StataCorp, College Station, Texas 77845 USA).

In order to observe the full-time series of the angle assessments, statistical parametric mapping (SPM) was performed. The two-tailed paired t-test was used to compare the ankle kinematics between sides. The comparison between the ankle kinematic angle of Thai adults and the reference values supplied by the motion analysis program were computed by two-tailed unpaired t-test at each time point of right and left gait cycles. SPM was implemented in MATLAB (R2020b, The MathWorks Inc) using open-source code (M.0.4.8, www.spm1d.org). The significance level was set at *α* = 0.05.

## Results

### Participant's demographic data

A total of 98 volunteers aged average of 28.6 ± 5.4 years, 62 (63.3%) of them were female. The mean height was 165.6 ± 7.6 cm and mean weight was 58.3 ± 9.2 kg. The mean BMI was 21.2 ± 2.0 kg/m^2^. Demographic data were presented in Table 1.

**Table 1:** Participants demographic data.

Characteristics	*N* = 98
Age (year), mean (SD)	28.6 (5.4)
Gender (*N*, %)	
Female	60 (61.2)
Male	38 (38.8)
Height (cm), mean (SD)	165.6 (7.6)
Weight (kg), mean (SD)	58.3 (9.2)
BMI (kg/m^2^), mean (SD)	21.2 (2.0)
Gait velocity (m/s), mean (SD)	1.1 (0.1)
Stride length (m), mean (SD)	1.2 (0.1)
Right step length (m), mean (SD)	0.6 (0.0)
Left step length (m), mean (SD)	0.6 (0.0)

SD = standard deviation.

### Ankle kinematics

Ankle kinematics were reported as ankle varus-valgus, ankle dorsiflexion-plantar flexion, foot progression, and ankle rotation. From Table 2, the right and left ankles were in the varus position at heel strike and mid-swing. For the sagittal and transverse planes, there was ankle dorsiflexion, external foot progression and ankle internal rotation entire gait cycle. The average ankle kinematics entire gait cycle ranged from −1.62 to 3.17 degrees for varus-valgus, 0.67 to 14.52 degrees for dorsiflexion-plantar flexion, −21.73 to −8.47 degrees for foot progression, and 5.22–9.74 degrees for ankle rotation (Table 3).

**Table 2:** The comparison of ankle kinematics between sides.

Ankle kinematics (degree)	Right (*N* = 98)		Left (*N* = 98)		*P-*value
	Mean	SD	Mean	SD	
Varus-valgus					
Heel strike	1.771	9.157	3.583	9.204	0.201
Mid-stance	−2.657	8.907	−0.571	9.030	0.082
Terminal stance	−1.609	9.830	0.587	9.996	0.102
Mid-swing	2.386	8.802	3.963	8.695	0.179
Dorsiflexion-plantar flexion					
Heel strike	0.818	3.269	0.522	3.119	0.433
Mid-stance	7.437	2.956	6.912	3.046	0.077
Terminal stance	14.626	3.047	14.404	3.065	0.494
Mid-swing	3.719	3.717	3.049	3.946	0.081
Foot progression					
Heel strike	−12.148	5.197	−10.772	6.147	0.004*
Mid-stance	−9.122	5.069	−7.818	6.201	0.011*
Terminal stance	−11.179	5.205	−9.718	6.380	0.005*
Mid-swing	−22.175	7.035	−21.280	7.467	0.119
Rotation					
Heel strike	5.437	6.444	5.463	5.111	0.968
Mid-stance	5.224	6.501	5.221	5.351	0.937
Terminal stance	5.297	6.239	5.668	5.087	0.559
Mid-swing	9.339	7.043	10.132	5.726	0.298

+ = ankle varus, dorsiflexion, internal rotation and internal foot progression; − = ankle valgus, plantar flexion, external foot progression and external rotation.

* Significant *P*-value < 0.05.

**Table 3:** The comparison between different reference data sets.

Ankle kinematics (degree)	Thai adults (*N* = 98)		Motion analysis Software		*P*-value
	Mean	SD	Mean	SD	
Varus-valgus					
Heel strike	2.677	7.676	−0.250	0.399	<0.001*
Mid-stance	−1.614	7.601	−0.869	0.563	0.186
Terminal stance	−0.512	8.371	−2.553	1.129	0.029*
Mid-swing	3.174	7.355	−0.189	0.316	<0.001*
Dorsiflexion-plantar flexion					
Heel strike	0.670	2.738	1.588	1.648	0.001*
Mid-stance	7.174	2.624	4.190	1.370	<0.001*
Terminal stance	14.515	2.602	12.716	1.241	<0.001*
Mid-swing	3.384	3.372	0.921	2.635	<0.001*
Foot progression					
Heel strike	−11.459	5.216	−16.359	3.965	<0.001*
Mid-stance	−8.469	5.118	−11.572	2.600	<0.001*
Terminal stance	−10.448	5.268	−13.406	2.559	<0.001*
Mid-swing	−21.728	6.686	−25.957	6.849	<0.001*
Rotation					
Heel strike	5.452	4.796	−8.661	5.040	<0.001*
Mid-stance	5.222	4.918	−12.095	4.338	<0.001*
Terminal stance	5.482	4.709	−10.743	3.524	<0.001*
Mid-swing	9.736	5.323	−7.081	4.715	<0.001*

+ = ankle varus, dorsiflexion, internal foot progression and internal rotation, − = ankle valgus, plantar flexion, external foot progression and external rotation.

* Significant *P*-value < 0.05.

### Side difference

**Figure 2:**
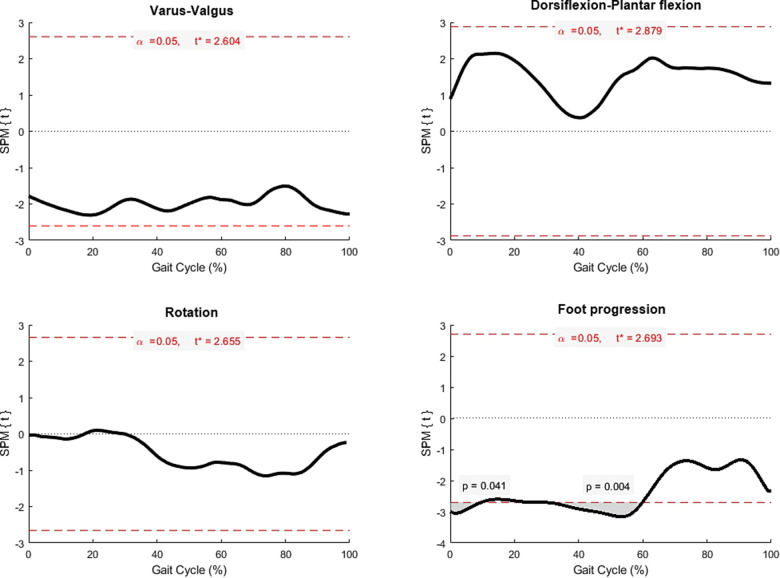
Statistical parametric mapping (SPM) comparing ankle kinematics between sides (black line), and grey area represented significant difference at points of time with *P* < 0.05.

The ankle kinematics were not statistically different between sides, except foot progression at the stance phase (*P* = 0.004 at heel strike, *P* = 0.011 at mid-stance, and *P* = 0.005 at terminal stance). Descriptive data of bilateral 3-dimension ankle kinematics were presented in Table 2. SPM analysis showed significantly different foot progression at initial contact (*P* = 0.041) and terminal stance (*P* = 0.004) between sides, Figure 2.

### Reference data

**Figure 3:**
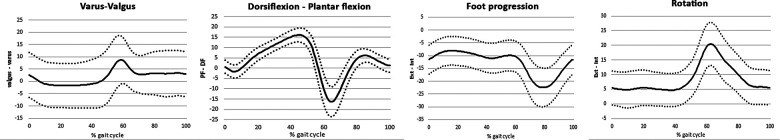
Ankle kinematic curves (degree) during gait cycle from our reference, mean (solid line) and standard deviation (dotted line), PF = plantar flexion, DF = dorsiflexion, Ext = external rotation, and Int = internal rotation.

**Figure 4:**
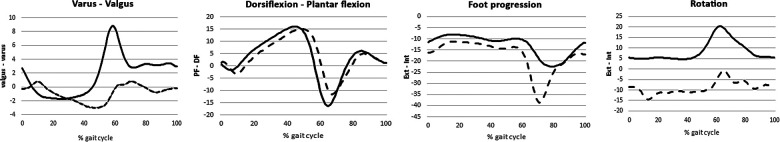
The comparison of ankle kinematics between our data (solid line) and the motion analysis reference (broken line), PF = plantar flexion, DF = dorsiflexion, Ext = external rotation, and Int = internal rotation.

**Figure 5:**
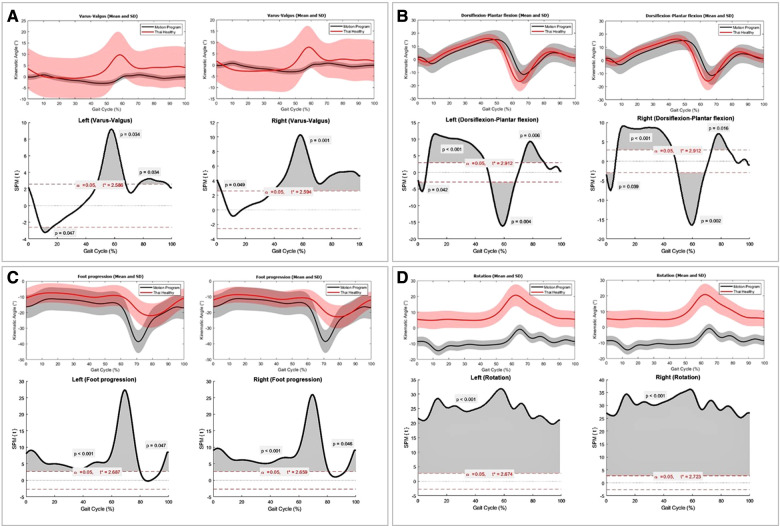
The comparisons of right and left ankle kinematics between Thai adults (red), the reference values of the motion analysis program (grey), mean (line) and standard deviation (shaded area). Statistical parametric mapping (SPM) demonstrated full-time series analysis of different ankle kinematics between two data sets (black curve). Grey areas under the black curve represented significant differences at gait cycle (%) point of time with *P* < 0.05. (**A**) varus-valgus, (**B**) dorsiflexion-plantar flexion, (**C**) foot progression, and (**D**) ankle rotation planes.

Inception data of ankle kinematics at heel strike, mid-stance, terminal stance, and mid-swing were summarized as graphs (Figure 3). Comparing to the reference of the motion analysis program, our ankle kinematics significantly increased ankle varus, dorsiflexion, internal rotation of foot progression and ankle rotation (*P* < 0.001), except ankle varus-valgus at mid-stance (*P* = 0.19) (Table 3 and Figure 4). Regarding SPM analysis stratified by sides, the ankle kinematic angle of ours significantly differed from the reference values supplied by the motion program in all planes (Figure 5). Significant *P*-value of each plane (Figure 5) were demonstrated as varus-valgus (left side: *P* = 0.047, 0.004, and 0.004; right side: *P* = 0.001), dorsiflexion-plantar flexion (left side: *P* = 0.042, <0.001, 0.004, and 0.006; right side: *P* = 0.009, < 0.001, 0.002, and 0.016), foot progression (left side: *P* < 0.001, and 0.047; right side: *P* < 0.001, and 0.046), and ankle rotation (*P* < 0.001 for both sides).

### Relationship with age, gender, and BMI

According to significant side difference in foot progression (Table 2), the right and the left side were taken into account by separately relating with age, gender and BMI. From Table 4, advanced age had significantly correlated with ankle internal rotation at mid-swing (beta coefficient 0.21, *P* = 0.039), and tended to have ankle valgus, plantar flexion with external foot progression. Male gender had approximately 5 degrees more external foot progression than the female group at heel strike, mid-stance, and terminal stance of the right (*P* = 0.002, 0.002, and 0.008, respectively) and the left sides (*P* = 0.006, 0.012, and 0.032, respectively). The relationship between BMI did not reach statistical significance with the ankle kinematics in all planes (Table 4).

**Table 4:** The relationship between ankle kinematics and age, gender, and BMI.

Ankle kinematics (degree)	Age (years)			Male gender			BMI (kg/m^2^)		
	Coefficient	95% CI	*P*-value	Coefficient	95%CI	*P*-value	Coefficient	95%CI	*P*-value
Varus-valgus									
Heel strike	−0.04	−0.32, 0.25	0.802	0.51	−2.66, 3.68	0.750	0.60	−0.15, 1.35	0.117
Mid-stance	0.04	−0.24, 0.32	0.784	0.32	−2.82, 3.46	0.840	0.64	−0.11, 1.39	0.092
Terminal stance	0.08	−0.23, 0.39	0.599	−0.08	−3.54, 3.38	0.964	0.62	−0.20, 1.45	0.136
Mid-swing	0.02	−0.25, 0.30	0.858	−0.21	−3.26, 2.83	0.889	0.55	−0.17, 1.27	0.134
Dorsiflexion-plantar flexion									
Heel strike	−0.06	−0.16, 0.05	0.287	0.88	- 0.23, 2.00	0.120	−0.01	−0.28, 0.26	0.938
Mid-stance	−0.017	−0.12, 0.08	0.729	0.99	−0.07, 2.06	0.067	−0.03	−0.29, 0.23	0.803
Terminal stance	−0.01	−0.11, 0.08	0.782	−0.16	−1.24, 0.91	0.767	0.09	−0.16, 0.35	0.475
Mid-swing	−0.08	−0.20, 0.05	0.213	0.77	−0.61, 2.16	0.272	−0.24	−0.58, 0.09	0.146
Right foot progression									
Heel strike	−0.05	−0.24, 0.15	0.634	−3.34	−5.38, −1.30	0.002*	−0.44	−0.95, 0.07	0.087
Mid-stance	−0.03	−0.22, 0.16	0.756	−3.18	−5.17, −1.18	0.002*	−0.39	−0.89, 0.10	0.116
Terminal stance	−0.05	−0.25, 0.14	0.599	−2.84	−4.92, −0.76	0.008*	−0.36	−0.87, 0.16	0.171
Mid-swing	−0.22	−0.48, 0.04	0.092	1.65	−1.24, 4.54	0.261	0.14	−0.56, 0.84	0.693
Left foot progression									
Heel strike	−0.03	−0.26, 0.19	0.781	−3.49	−5.93, −1.05	0.006*	−0.37	−0.98, 0.24	0.228
Mid-stance	−0.07	−0.30, 0.16	0.553	−3.19	−5.67, −0.70	0.012*	−0.34	−0.96, 0.27	0.271
Terminal stance	−0.10	−0.34, 0.14	0.393	−2.83	−5.40, −0.25	0.032*	−0.32	−0.95, 0.31	0.314
Mid-swing	−0.22	−0.50, 0.05	0.109	1.15	−1.94, 4.23	0.462	−0.12	−0.86, 0.62	0.753
Rotation									
Heel strike	0.13	−0.05, 0.31	0.147	0.38	−1.60, 2.36	0.705	0.40	−0.07, 0.87	0.093
Mid-stance	0.17	−0.01, 0.35	0.062	0.44	−1.59, 2.47	0.668	0.28	−0.20, 0.77	0.248
Terminal stance	0.14	−0.04, 0.31	0.121	0.06	−1.88, 2.01	0.948	0.15	−0.31, 0.63	0.505
Mid-swing	0.21	0.01, 0.40	0.039*	−8.67	−3.06, 1.33	0.434	0.46	−0.06, 0.98	0.085

* Significant *P*-value < 0.05.

## Discussion

This prospective study established normative 3-dimension ankle kinematic references including foot progression for Thai adults. We found significant difference between our data and the motion analysis reference in all planes, except ankle varus-valgus in mid-stance. Foot progression differed between sides and related well with gender. Age was significantly associated with ankle rotation at mid-swing, whereas there was no significant relationship between ankle kinematics and BMI.

Ankle kinematics varied with regards to racial, cultural, and ethnic properties. Im et al. (11) compared gait characteristics between Korean and Western young adults. Most of motion patterns and excursions were similar but ranges of angles were different. Average ankle motion of Koreans (Motion Analysis) was −1.9 to 3.3, −17.5 to 10.8 and −17.3 to −3.5 degrees for inversion-eversion, dorsiflexion-plantar flexion, and rotation, respectively (11). Although the mean ankle kinematics from Western's studies (Elite, VICON) were −3.2 to 9.2 (21) for inversion-eversion, and −22.6 to 10.9 (21) and −14.0 to 11.5(18) for dorsiflexion-plantarflexion, and −19.0 to −5.0 for rotation (18), our ankle motion obviously showed more varus, dorsiflexion, internal foot progression, and ankle internal rotation (Table 5) when compared to the motion analysis program and other studies (11, 18, 21). For ankle dorsiflexion, it resembled Cho et al (17), and Brockett et al (2), but was contradictory to the other reports of plantar flexion during the first rocker (11, 16, 18, 21). Our data showed striking ankle internal rotation (min-max difference 4.68, average total rotation 20.48, standard deviation 8.46), while the study from Korea (11) using the same motion analysis system reported min-max difference of −17.3, average total rotation −3.50 (standard deviation 13.8). This emphasizes that culture, anthropometry, and other factors might contribute to these differences (9).

**Table 5:** The comparison of ankle kinematics between studies.

Variables	Our study **(***N* **= **98**)**	Im et al**.** (11) 2006 **(***N* **= **32**)**	Cho et al**.** (17) 2004 **(***N* **= **98**)**	Benedetti et al**.** (21) 1998 **(***N* **= **20**)**	Kadaba et al**.** (18) 1990 **(***N* **= **40**)**
Setting	Thailand	Korea	Korea	Italy	USA
Age (year), mean (range)	28.6	Female = 25	Female = 22.9	43	NA
	(18–40)	Male = 24.1	Male = 23.5	(20 −72)	(18–40)
Measurement system	Motion Analysis	Motion Analysis	VICON 370	Elite system	VICON
	Santa Rosa, USA	Santa Rosa, USA	Oxford, UK	Milano, Italy	Oxford, UK
Gait velocity (m/s), mean (SD)	1.12 (0.1)	1.14 (0.1)	1.16 (0.1)	1.26 (0.2)	1.34 (0.2)
Stride length (m), mean (SD)	1.20 (0.1)	1.27 (0.1)	1.21 (0.1)	1.40 (0.2)	1.41 (0.1)
Total varus-valgus (degree)					
Min-max difference	−1.7	−1.9	NA	−3.2	NA
Mean (SD)	8.8 (1.8)	3.3 (5.2)		9.2 (13.4)	
Total dorsiflexion-plantar flexion (degree)					
Min-max difference	−16.3	−17.5	−12.0	−22.6	−14.0
Mean (SD)	16.1 (3.6)	10.8 (28.3)	19.5 (31.5)	10.9 (33.5)	11.5 (25.5)
Total rotation (degree)					
Min-max difference	4.7	−17.3	NA	NA	−19.0
Mean (SD)	20.5 (8.5)	−3.5 (13.8)			−5.0 (14.0)

NA = not available; SD = standard deviation; + = ankle varus, dorsiflexion, internal rotation and internal foot progression; − = ankle valgus, plantar flexion, external foot progression and external rotation.

Ankle kinematics may vary between sides. The previous study demonstrated 3–5 degrees of ankle dorsiflexion-plantar flexion differences (22). Our study found that only foot progression at the stance phase was 1–2 degrees different between sides, and associated with gender. The right foot was significantly more external rotated that the left one. Males demonstrated external foot progression or out-toeing about 5 degrees more than females. Men's habits or lifestyles may alter foot progression at the stance phase. Cibulka et al (23) reported an average foot progression in men was 3 degrees more external rotation than in women. The authors proposed the tibiofemoral external torsion might contribute to external foot progression, whilst hip internal rotation was associated with internal foot progression. However, other studies concluded that gender had no effect on ankle kinematics without considering the foot progression (11, 16–18).

Participants' age ranges between 18 and 40 years old usually express the mature gait pattern, with less variability when compared with children, and are unaffected from degenerative changes when compared to the elderly. Our study demonstrated significant association between age 18 and 40 years and ankle rotation at mid-swing (0.21-degree per increment 1 year of age). The ankle kinematics in other planes, especially dorsiflexion-plantar flexion was not significantly different in this young age range. Praditpod et al (16) conducted the sagittal plane gait analysis in healthy Thais, aged 20–69 years old, using the VICON system. The authors found that advanced age was negatively correlated with ankle dorsiflexion-plantarflexion at toe-off, without correlation at heel contact, foot flat, mid-stance and heel off. According to restraint strength of plantar flexor, the elderly tended to have lesser plantar flexion, and ankle moment at push-off than the younger age (14, 15). BMI did not contribute to ankle joint kinematics changes, but high body mass tended to increase ankle varus, plantar flexion, external foot progression, and ankle internal rotation. Recent study compared the difference of BMI groups on the change of gait kinematics by using the H-Gait system with inertial sensors to record gait data (24), they showed no difference of ankle kinematics between BMI 18.5 and 25 kg/m^2^. This finding was similar to our study, even though we used Motion analysis software for gait analysis.

Apart from different gait analysis systems, discrepancy of measurement may indicate variation of ankle kinematics within population. Regarding an ankle joint complex, gait analysis cannot separately measure rotation and other movements of talar joints (2). Skin motion artifact affects joint movement in the frontal and transverse planes (21). The amount of discrepancy among adduction–abduction, flexion–extension, and internal–external rotation are roughly to 50%, 10% and 100%, respectively (21). In addition, deviations between intra- and inter-examiner can occur in the position of marker on anatomical landmark which has a difference about 6–21 and 13–25 millimeters, respectively (22). Different marker models might influence ankle kinematic results. Kadaba and colleague designed the marker system of Helen Hayes and found significant errors in the ankle inversion-eversion and rotation angles throughout the gait cycle (18). Although our study referred the Helen Hayes marker set from previous studies (18), we modified it by adding the medial knee and the medial malleolus markers. We ensured perfect marker positions since all of our participants had normal BMI and well-prominent bony landmarks. All 29 markers, including hidden medial knees, were clearly detected by digital cameras and well-connected together in the program.

In light of limited evidence of the normal gait pattern in Thailand, we are able to define the reference values of gait in Thai adults. The strengths of this study are objective measurements of ankle kinematics using standardized Motion Analysis Software, adequate sample size, and appropriate statistical analysis. Our study established kinematics of the ankle joint complex which is involved in motion of the foot and ankle in ankle varus-valgus, ankle dorsiflexion-plantar flexion, foot progression and ankle rotation. Moreover, we added the factor of BMI accompanied with ankle motion of healthy subjects in addition to previous evidences. This reference is useful to understand ankle movement pattern of Thai adults and may use to detect gait problem in patients with ankle injury and neuromuscular problems in the future. Moreover, factors related to ankle kinematics might predict some pathology, i.e., ankle internal rotation in advanced age. The limitations of our study are (1) unable to generalize for adults older than 40 years; (2) inconsiderate hip and knee kinematics; and (3) unavailable baseline characteristics of the motion analysis reference data to be compared. Race and culture may affect gait pattern such as cadence and joint moment (17). Walking speed is a main factor influences the ankle-foot complex plantarflexion at toe-off and during swing phases (11, 21). Further study of all hip, knee, and ankle kinematics is needed to provide reference values of complete gait analysis. Also, the effect of gait characteristics on joint kinematics as well as race and cultural background should be investigated. According to Thai traditionally culture, we hypothesize that females are most familiar with a sit one leg tucked back to one side or side monk. Similar to W-sitting with one leg, this posture promotes femoral internal rotation. This may lead to internal rotation of the foot during walking and decreasing external rotated foot progression, particularly in females.

## Conclusion

This study established the normal reference ankle kinematics of Thai adult, aged 18–40 years, which is significantly different from that of the motion analysis program in all three planes. External foot progression was appeared on the right more than the left side, and males more than females. The greater age was correlated with the ankle internal rotation. This new reference value may be suitable for Thai adults, and further population-based study is required to estimate reference values for all age groups.

## Data Availability

The original contributions presented in the study are included in the article/Supplementary Material, further inquiries can be directed to the corresponding author/s.
